# Vitexin as a Potential Antidysmenorrheic Agent: Development of a ZIF-8-Based Immediate-Release System and Evaluation via In Vivo and In Silico Approaches

**DOI:** 10.3390/biomedicines13112602

**Published:** 2025-10-24

**Authors:** José Marcos Teixeira de Alencar Filho, Ana Rita de Sousa França, Luana Beatriz Rocha da Silva, Pedrita Alves Sampaio, Emanuella Chiara Valença Pereira, Ademar Rocha da Silva, Milenna Victória Valentim de Oliveira Alencar, Tarcísio Cícero de Lima Araújo, Pedro Modesto Nascimento Menezes, Salvana Priscylla Manso Costa, Ighor Costa Barreto, Fabrício Souza Silva, Edigênia Cavalcante da Cruz Araújo, Edilson Beserra de Alencar Filho, Larissa Araújo Rolim

**Affiliations:** 1 Department of Bromatological Analysis, Faculty of Pharmacy, Universidade Federal da Bahia (UFBA), Barão de Jeremoabo Street, 147, Ondina Campus, Salvador 40170-115, BA, Brazil; 2 Colegiado de Ciências Farmacêuticas, Universidade Federal do Vale do São Francisco (UNIVASF), José de Sá Maniçoba Avenue, Downtown, Petrolina 56304-917, PE, Brazil; francaanarita@gmail.com (A.R.d.S.F.); luana.beatrizr@discente.univasf.edu.br (L.B.R.d.S.); fabricio.souzasilva@univasf.edu.br (F.S.S.); edigenia.araujo@univasf.edu.br (E.C.d.C.A.); edilson.beserra@univasf.edu.br (E.B.d.A.F.); larissa.rolim@univasf.edu.br (L.A.R.); 3 Postgraduate Program in Biological Health Sciences, Universidade Federal do Vale do São Francisco (UNIVASF), José de Sá Maniçoba Avenue, Downtown, Petrolina 56304-917, PE, Brazil; 4 Central de Análises de Fármacos, Medicamentos e Alimentos (CAFMA), Colegiado de Ciências Farmacêuticas, Universidade Federal do Vale do São Francisco (UNIVASF), José de Sá Maniçoba Avenue, Downtown, Petrolina 56304-917, PE, Brazil; sampaiopedrita@gmail.com (P.A.S.); emanuella.chiara@gmail.com (E.C.V.P.); milennaalnc_@outlook.com (M.V.V.d.O.A.); tarcisiocla62@gmail.com (T.C.d.L.A.); 5 Postgraduate Program in Medicine and Human Health, Escola Bahiana de Medicina e Saúde Pública, Dom João VI Avenue, 275, Salvador 40290-000, BA, Brazil; adeemarrocha@gmail.com; 6 Laboratório de Farmacologia Experimental (LAFEX), Colegiado de Ciências Farmacêuticas, Universidade Federal do Vale do São Francisco (UNIVASF), José de Sá Maniçoba Avenue, Downtown, Petrolina 56304-917, PE, Brazil; p.modesto89@gmail.com; 7 Department of Pharmacy, Universidade do Estado da Bahia (UNEB), Silveira Martins Street, 2555, Cabula, Salvador 41150-000, BA, Brazil; salvanacosta@gmail.com; 8 Environmental Coordination, Universidade Federal da Bahia, Barão de Jeremoabo Street, Ondina Campus, Salvador 40170-115, BA, Brazil; ighor.barreto@ufba.br

**Keywords:** physicochemical characterization, MOF, primary dysmenorrhea, in vitro release, molecular docking and dynamics

## Abstract

**Background/Objectives**: Primary dysmenorrhea is a prevalent condition that causes severe uterine cramps in women worldwide. The objective of this work was to synthesize and characterize a novel immediate-release system using vitexin and ZIF-8, and to evaluate its pharmacological action in a model of primary dysmenorrhea. **Methods**: A 2^2^ full factorial design guided the synthesis of the system. Physicochemical characterization was performed by FT-IR, TG, DSC, SEM, XRD, and in vitro release tests. Pharmacological activity was assessed in an oxytocin-induced dysmenorrhea model in mice. In addition, in silico molecular docking and molecular dynamics simulations were conducted to explore the potential mechanism of action of vitexin. **Results**: Optimal yield and loading capacity were achieved at the high levels of the factorial design. Characterization analyses confirmed the successful formation of the vitexin@ZIF-8 (VIT@ZIF-8) system. The release study demonstrated a markedly enhanced dissolution rate of vitexin. Both isolated vitexin and VIT@ZIF-8 reduced abdominal writhing when administered orally at 3 and 30 mg/kg, while intraperitoneal activity was observed only at 30 mg/kg. Computational analyses revealed favorable interactions of vitexin with aldose reductase (AKR1C3), suggesting this enzyme as a potential molecular target in dysmenorrhea. **Conclusions**: The VIT@ZIF-8 system represents a promising strategy to improve the dissolution profile of vitexin, although pharmacological activity in this model was not superior to the isolated compound. The combined in vivo and in silico evidence supports vitexin as a potential antidysmenorrheic agent, possibly through modulation of AKR1C3. These findings open avenues for future studies addressing the molecular pathways of vitexin and for the development of novel therapeutic approaches for primary dysmenorrhea.

## 1. Introduction

Primary dysmenorrhea is a painful uterine contraction caused by an endometrial tear. The pain caused by dysmenorrhea begins a few days before menstruation and persists for 48 to 72 h, with cramp-like pain that can extend to the leg muscles, representing one of the most common complaints of adolescents and mature women. It usually comes with a wide range of physical symptoms, such as headaches, dizziness, fatigue, diarrhea, cramps, sweating, and is sometimes incapacitating for daily activities [1,2,3]. It can be classified as primary or secondary dysmenorrhea; the primary one is menstrual pain associated with ovular cycles in the absence of pathological findings and which affects 20–90% of women of reproductive age [4,5]. Its etiology is not well understood, but most symptoms can be explained by the production of uterine prostaglandins (PGs), particularly PGF_2α_, which are released by disintegrating endometrial cells and act by stimulating uterine muscle contractions [6,7].

The enzyme cyclooxygenase-2 (COX-2) is classically attributed to being responsible for the synthesis of prostaglandins. However, the scientific literature has highlighted the role of the human aldose reductase enzymes (as AKR1C3) in the synthesis of PGF_2α_ in the endometrium, especially in inflammatory conditions such as dysmenorrhea [8,9,10]. Studies have shown that aldo-keto reductases act as a potent PGF_2α_ synthetase, contributing significantly to the increase in this prostaglandin in human endometrial tissues. This increase is associated with the intense pain characteristic of primary dysmenorrhea, due to the vasoconstrictor and uterine contraction-stimulating action of PGF_2α_. Furthermore, inhibition of aldo-keto reductases by compounds such as ponalrestat has been shown to reduce PGF_2α_ production, suggesting therapeutic potential in the control of menstrual pain. These findings position AKR1C3 as a promising target for the development of novel approaches to the treatment of dysmenorrhea [8,9].

Several types of treatment have been reported to reduce the symptoms of dysmenorrhea, which may include non-specific non-steroidal anti-inflammatory drugs (NSAIDs), medicinal plants, acupuncture, heat therapy, aromatherapy, yoga practice, and diet, among others [11,12,13,14,15]. NSAIDs are considered the primary treatment for primary dysmenorrhea, with mefenamic acid, diclofenac, ibuprofen, and naproxen as the main representatives, but they commonly cause side effects that include indigestion, diarrhea, peptic ulcers, dizziness, and headaches [6,16]. The appearance of these unwanted effects has led many women to look for alternative and complementary therapies that are effective in relieving the painful symptoms caused by cramps [17,18,19].

From this perspective, natural products have stood out due to the fact that access by traditional communities and those far from urban centers is easier than synthetic medicines, and generally with lower acquisition costs. The scientific literature already reports the mechanisms by which some plants act to reduce the symptoms of primary dysmenorrhea, such as fennel (*Foeniculum vulgare* Mill), chamomile (*Matricaria chamomilla* L.) and ginger (*Zingiber officinale* Roscoe), which are effective for their antispasmodic, analgesic and anti-inflammatory effects, due to the presence of a range of phytochemical constituents such as monoterpenes, sesquiterpenes, phenolic acids and flavonoids [11,18,20,21].

In this context, vitexin (apigenin-8-C-β-*D*-glucopyranoside) is a glycosyl flavone found in several medicinal plants such as *Jatropha mutabilis* (Pohl) Baill [22]. *J. mutabilis* is an endemic species of the Brazilian caatinga biome, popularly known as “pinhão-de-seda” and is used by local communities as a depurative and for intestinal constipation. Few studies have been published addressing its chemical and pharmacological potential; however, it is believed that this species is a promising source of vitexin, a chemical marker widely present in the *Jatropha* genus [22,23].

Vitexin has stood out due to its previously described pharmacological actions, mainly related to inflammation, pain, and diseases related to oxidative stress [24,25]. Vitexin (10 mg/kg, ip) was able to inhibit pain associated with inflammation and also inhibited 91% of the acetic acid-induced abdominal writhing response and pain-like behavior. As the possible mechanism, vitexin could prevent the reduction of glutathione levels, the potential of iron-reducing capacity, and the free radical scavenging capacity, as well as inhibit the production of hyperalgesic cytokines (TNF-α, IL-1β, IL-6, and IL-33) and increase levels of the antihyperalgesic cytokine IL-10 [26].

However, vitexin has low aqueous solubility (7.62 μg/mL), which could be a serious problem related to its dissolution and gastrointestinal absorption [23]. Strategies can be used to improve the release, dissolution, and absorption of low solubility drugs, such as reducing particle size, creating salts or esters, and, more recently, the creation of immediate release systems and inclusion complexes [27,28]. Therefore, metal–organic frameworks (MOFs), which are porous polymers composed of an organic part and a metallic part, can be alternatives to improve therapeutic profiles of drugs with solubility problems [29,30].

Among MOFs, ZIF-8 has been extensively studied for transporting drugs and drug candidates due to some of its physical properties, such as pore dimensions (gauge size), high surface area (1.947 m^2^ g), high thermal stability (up to 550 °C), pH-responsive dissolution behavior, and relative biocompatibility [31,32,33]. Formed by tetrahedral zinc with 2-methylimidazole, this MOF has been studied for the delivery of numerous substances, mainly for the treatment of cancer [34,35].

Despite extensive studies on ZIF-8 as a drug carrier, no investigations have reported its application in developing an immediate-release system for vitexin, nor have preclinical evaluations of this flavonoid been conducted in models of primary dysmenorrhea. Considering the high prevalence and disabling impact of this condition, coupled with the limitations of current NSAID-based therapy, there is a clear need for innovative, fast-acting, and safer alternatives. Therefore, this study aimed to synthesize and physicochemically characterize a novel vitexin@ZIF-8 (VIT@ZIF-8) immediate-release system, evaluate its pharmacological potential in an oxytocin-induced murine model of primary dysmenorrhea, and investigate the possible molecular mechanisms of action of vitexin through in silico docking and molecular dynamics simulations. By combining experimental and computational approaches, this work seeks to provide new insights into the therapeutic value of vitexin and establish a foundation for the development of alternative strategies for the management of primary dysmenorrhea.

## 2. Materials and Methods

### 2.1. Planning and Synthesis of Vitexin@ZIF-8 System (VIT@ZIF-8)

For the synthesis of the release system from vitexin and ZIF-8, a 1:2 ratio between Zinc (Zn) and 2-methylimidazole (2-MeIM) was used in a complete factorial design 2^2^. The first factor considered was the molar proportion of vitexin in relation to the reagents for obtaining ZIF-8, and the second factor was the reaction time, as can be seen in Table 1. Therefore, 4 conditions of synthesis were established, according to Table 2. Zn and 2-MeIM were dissolved in distilled water and vitexin was dissolved in distilled water and 10% ethanol. All reactions were carried out at rest and at room temperature.

Solutions were prepared at the following concentrations: ZnSO_4_.7H_2_O containing 80 μM Zn, 2-MeIM at 160 μM, and vitexin at 4 μΜ (low level) or 8 μΜ (high level). Thus, 10 mL of the 2-MeIM solution was placed in a 100 mL beaker and 10 mL of the vitexin solution was added with slight manual stirring. Then, 10 mL of the ZnSO_4_.7H_2_O solution was added, where the formation of a yellow precipitate was immediately observed. The reactions took place at the times pre-determined by the experimental design and immediately afterwards, they were filtered through a quantitative filter and the material was left to dry in an oven at 50 °C for 6 h. Subsequently, a sample of ZIF-8 was synthesized using the same 24 h reaction time for comparison of characterization tests.

At this stage, two dependent variables were considered and analyzed: the yield of the synthesis process and the rate of loading (encapsulation) of vitexin into the system. The yield was calculated from the equation x = 100 × y/TMY, where x is the percentage of the observed yield, y is the VIT@ZIF-8 system mass obtained, and TMY is the theoretical maximum yield. The loading capacity was obtained by direct quantification of vitexin after opening the system by acidification. The Statistica 7.0 software was used to construct the response surface graphs. The unpaired Student *t* test was used to verify significant differences between the yields and incorporation rates obtained.

### 2.2. Characterization Tests

All characterization tests were carried out with vitexin and ZIF-8, but for the VIT@ZIF-8 system, characterization was only carried out on the sample obtained in the condition considered to be the one with the highest yield and highest loading capacity.

#### 2.2.1. Fourier Transform Infrared Absorption Spectroscopy (FT-IR)

The infrared spectra of the analyzed samples were obtained using potassium bromide (KBr) pellets as a solid support. To prepare the tablet, approximately 1 mg of the sample was used for 100 mg of KBr, which was crushed until a fine powder was obtained. The mixture was then subjected to pressure of 78.5 KN, using a hydraulic press (Shimadzu^®^, Kyoto, Japan) for a period of 10 min. After obtaining the KBr tablet, it was analyzed on a spectrometer (IRTracer-100, Shimadzu^®^, Kyoto, Japan) in the region between 4500 and 600 cm^−1^, with 45 scans and a resolution of 4 cm^−1^ [36].

#### 2.2.2. Thermal Analysis: Differential Scanning Calorimetry (DSC) and Thermogravimetry (TG)

To obtain the DSC curves, the Shimadzu^®^ Calorimeter (DSC-60, Kyoto, Japan) was used, connected to the Shimadzu^®^ TA-60WS software and 2 mg of each sample was used, placed in an aluminum crucible. The device was calibrated with a heating rate of 10 °C/min, with a temperature variation of 0–300 °C and a nitrogen flow of 50 mL/min. Indium (156.4 °C) and Zinc (419.5 °C) were used to calibrate the temperature scale and the enthalpy response of the equipment. The TG curves were obtained using the Shimadzu^®^ thermobalance (TGA Q60, Kyoto, Japan), where 5 mg of the samples were weighed in a platinum crucible. The device was calibrated with a heating rate of 10 °C/min, with a temperature variation of 0–600 °C and a nitrogen flow rate of 100 mL/min. All analysis processes were preceded by spraying, screening, and quantitative weighing of the sample [37].

#### 2.2.3. Scanning Electron Microscopy (SEM)

In SEM analyses, the samples were dispersed on carbon tape fixed to the support (stub). They were then metallized with atomic gold in a metallizer (Quorum model Q150R ES, Laughton, UK) with a current of 15 mA for 5 min with a tooling factor of 2.30. The samples were analyzed using a scanning electron microscope (Vega3 SEM model, Tescan, Brno, The Czech Republic) coupled to an energy dispersive X-ray spectroscopy (EDS) analysis system [38]. The images obtained by scanning electron microscopy (SEM) were analyzed using the ImageJ software (version 1.53k, National Institutes of Health, Bethesda, MD, USA). The scale was manually calibrated based on the reference bar present in the image. The particles were segmented by binary thresholding and analyzed using the “Analyze Particles” command, excluding objects at the edge of the image. The areas of the particles (in µm^2^) were extracted, and the equivalent diameter was calculated using the formula D=√4Aπ. The data were exported to a spreadsheet, where the descriptive metrics were calculated: mean diameter, median, and standard deviation [39,40].

#### 2.2.4. X-Ray Diffractometry (XRD)

Diffraction patterns of samples were obtained using an X-ray diffractometer (Shimadzu^®^ XRD 6000, Kyoto, Japan). The test used a voltage of 40 kV and a current of 30 mA. Diffraction patterns were performed ranging from 5 to 90°, with a step of 0.02° and an angular speed of 2 degrees/min. The divergence and scattering slits used were 1° and the reception slit was 0.30 mm, with CuKa radiation being used. Data were analyzed using the Bragg Equation: 2 × d. sinθ = n × λ, where: d = basal spacing; sin θ = sine of the theta angle; n = constant; and λ = copper wavelength [38].

#### 2.2.5. In Vitro Release Test

In a beaker, 200 mL of phosphate buffer (pH 7.2) and 10 mg of vitexin or a sufficient amount of VIT@ZIF-8 that was equivalent to 10 mg of vitexin were added, and kept under magnetic stirring at 200 rpm. Aliquots were taken at times 1, 15, 30, 60, 90, 120, 180, 240, 300, 360, 420, and 480 min for quantification of soluble vitexin, using the HPLC-DAD methodology previously described. The experiment had a controlled temperature at 37 °C, carried out in triplicate, and with the results expressed as mean ± standard deviation for the % of vitexin released [36].

### 2.3. Evaluation of the Effect of Vitexin and the VIT@ZIF-8 on Abdominal Contortions in Mice in an Experimental Model of Primary Dysmenorrhea

All procedures were conducted in accordance with ethical principles in animal experimentation, after approval of the project by the Ethics Committee on the Use of Animals (CEUA) of the Federal University of Vale do São Francisco (UNIVASF) (protocol # 0005/250521). All researchers involved in the experiments were aware of the allocation of animals into control and test groups.

Virgin mice aged between 6 and 8 weeks, weighing between 30 and 40 g, were used to induce the experimental model of primary dysmenorrhea, as described by Yang et al. [41]. All animals came from the central vivarium of the Federal University of Vale do São Francisco (UNIVASF), Petrolina, Brazil. Before the sample administration procedure, the animals were fasted for 12 h. Sixteen (16) groups, containing six (6) mice each, were maintained with estradiol benzoate injection (1 mg/kg by day) intraperitoneally (IP) for 3 consecutive days and on the fourth day the animals were treated orally (VO) or IP with phosphate buffer (pH 7.2) 10 mL/kg (negative control), vitexin or VIT@ZIF-8 (3, 10 and 30 mg/kg), and mefenamic acid 90 mg/kg (positive control). Thirty min after treatment, each mouse was injected with 0.4 U of oxytocin, and abdominal contortions (contraction of the abdominal wall, pelvic rotation, and stretching of the hind legs) were recorded for the subsequent 30 min. The percentages of inhibition of abdominal writhing after treatment with vitexin and VIT@ZIF-8 were calculated in comparison with the negative control group. After the experimental procedures, the animals were euthanized with a lethal injection of thiopental (100 mg/kg). For statistical analysis, the Kruskal–Wallis test was used, followed by Dunn’s post-test, with the “vitexin” test group and the “VIT@ZIF-8” test group being compared separately to the negative control group. To check whether there is a difference between the “vitexin” group and the “VIT@ZIF-8” group, the Mann–Whitney test was used.

### 2.4. Molecular Docking and Molecular Dynamics Calculations

Molecular docking is a widely used computational tool in drug research, enabling, among other things, the production of optimized complexes that allow the evaluation of interactions between small molecules and macromolecular targets [10]. In addition, Molecular Dynamics simulations allow for obtaining the temporal trajectory of molecular systems, such as the complexes obtained by docking, providing valuable improvements such as the possibility of free solvation by water, visualization of dynamic properties, and computation of dynamic energies of stabilization. It thus seeks to close an in silico cycle by more faithfully incorporating the aspects of multiple states of a receptor and induced docking. Thus, the molecular mechanism of action related to the antidysmenorrheal activity of vitexin was investigated via Docking in the enzymes type 2 cyclooxygenase (COX-2) [42] and aldose reductase [43], both related to the production of prostaglandins in the uterine processes investigated. A crystallographic complex of the drug used in the experimental pharmacological section (mefenamic acid) is available bound to the AKR1C3 isoform. Then, this protein was selected for comparative analysis. The following paragraphs detail the processes carried out.

Initially, the vitexin structure was obtained in SMILES format, using the SwissTargetPrediction platform—www.swisstargetprediction.ch (accessed on 10 Janurary 2025). This is a web-based tool designed to predict the most probable protein targets for small molecules. It employs a ligand-based approach, utilizing both 2D and 3D similarity measures to compare the input molecule with a curated database of known active compounds [44]. By identifying structurally similar molecules with established target information, SwissTargetPrediction infers potential targets for the query compound. In the case of vitexin, there was a high degree of probability associated with the enzyme aldose reductase, narrowing our hypotheses of mechanism of action in dysmenorrhea to this enzyme.

The molecular targets used were obtained from the RCSB PDB online database [45], with the codes 5IKR (COX-2 with mefenamic acid), 8JP1 (AKR1C3 with a flavonoid molecule–liquiritigenin), and 3R43 (AKR1C3 with mefenamic acid). Fortunately, obtaining crystallographic data for the two proteins mentioned in the introduction (COX-2 and AKR1C3) allows us to perform a comparative analysis, in addition to the fact that both are originally complexed with the standard drug from the pharmacology section (mefenamic acid). We also found the same isoform of aldose reductase complexed with a generic flavonoid, which also prompted us to perform another comparative analysis, considering that vitexin is in the same class. In in silico molecular analyses, the more data, experimental models, and comparative analyses available, the stronger the content of the evidence generated.

The enzymes and their respective original ligands were prepared in the UCFS Chimera-1.19 software [46], with the water molecules being removed and the hydrogen being added. Using the APBS software—https://server.poissonboltzmann.org/ (accessed on 15 Janurary 2025), the pH of the proteins was adjusted to 7.4, similar to that observed under physiological conditions of the human body. The vitexin 3D structure was obtained from the PubChem platform, previously minimized by the Molecular Mechanics Method (MM6) [47].

Redocking and docking were performed using the Autodock Tools-1.5.7 and Autodock Vina-1.5.6 software [48,49]. Redocking was performed for each crystallographic complex in order to validate the accuracy and reliability of the method employed, testing different sizes of grid boxes centered on the original ligands. Each box geometry took into account the best RMSD (root mean square deviation) value found after systematic tests. The spacing between the grid box points was 1 Å. The grid box location (x, y, z coordinates) for calculations in Autodock Vina was centered on each respective co-crystallographic ligand of each complex studied. In turn, the number of points in the x, y, and z dimensions was defined for each case according to the best redocking result (RMSD < 2.0 Å). These data were replicated for each protein during the docking assays with vitexin.

Molecular dynamics (MD) simulations were performed using GROMACS version 2019.2 [50]. On a Linux/Ubuntu workstation, to evaluate the structural and energetic stability of ligand–protein complexes obtained after dockings. Ligand topologies were generated using the automated topology builder (ATB) [51] online platform, employing the GROMOS 54a7 force field [52] for all components. Each complex was placed in the center of a cubic simulation box, which was then solvated using the SPC (simple point charge) [53] water model. To ensure system neutrality and physiological conditions, counter ions were added until a final ionic concentration of 0.15 M was achieved.

An initial energy minimization was carried out to remove any steric clashes or high-energy contacts. This step was followed by two equilibration phases under periodic boundary conditions: NVT (constant number of particles, volume, and temperature) ensemble for 100 ps; NPT (constant number of particles, pressure, and temperature) ensemble for 100 ps. During both equilibration phases, position restraints were applied to the ligand to maintain its orientation within the binding site.

The production MD simulation was run for 250 nanoseconds (ns) under a temperature of 310 K and a pressure of 1 atm. The first 20 ns of the trajectory were considered as part of the extended equilibration and can then be excluded from any formal analysis. The last 10 ns of the trajectory were extracted for average binding energy calculations using the g_mmpbsa-1.6 tool [54], and frames were sampled at 100 ps intervals.

Graphs of RMSD (root mean square deviation in Å) of alpha carbons, RMSD of ligands, minimum ligand–protein distances, as well as the number of ligand–protein hydrogen bonds formed during the simulation were obtained. The curves of these data were smoothed with the moving averages of the original values. The graphs were generated in Python-3.10 via matplotlib-3.2.2 on a Linux/Ubuntu-20.04 environment.

### 2.5. In Silico ADME Predictions

The structure of vitexin was submitted to the SwissADME [55] web server—http://www.swissadme.ch (accessed on 6 October 2025)—for prediction of physicochemical properties and descriptors related to absorption, distribution, metabolism, and excretion (ADME) profiles. SwissADME is a free online tool developed by the SIB Swiss Institute of Bioinformatics that implements multiple predictive models for drug-likeness and pharmacokinetics. The molecule was represented in canonical SMILES format and was pasted into the input window. The “Run” command was executed to perform the calculations and the results were promptly presented.

## 3. Results and Discussion

### 3.1. Synthesis of the VIT@ZIF-8 System and Determination of the Loading Capacity

The Supplementary Material contains the structural characterization of vitexin, where the ^1^H and ^13^C NMR chemical shifts can be observed in Table S1 [56,57,58,59].

**Figure 1:**
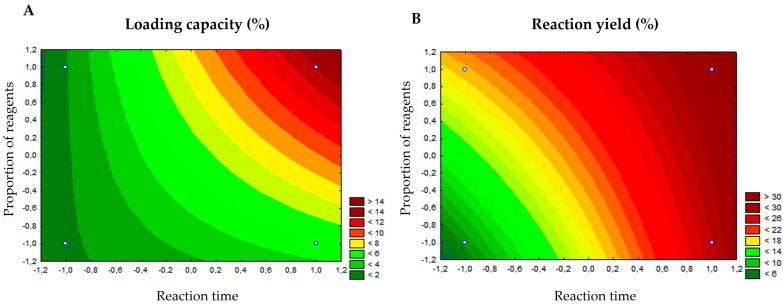
Response surface graph for the analyzed dependent variables. Legend: (**A**)—loading capacity; (**B**)—reaction yield. Both axes of figures A and B (x–Reaction time, and y–Proportion os reagents) go from −1.2 to +1.2 with an increment of 0.2. The time was counted in hours. The loading capacity in the Figure A expressed in the graph is represented from −2 to 16%, and the reaction yield in the Figure B is expressed from 0 to 35% with an increment of 5.

Considering that both the yield for obtaining drug delivery systems and loading capacity are important parameters for the analysis and technological production of this type of input, these two characteristics were then selected as dependent variables, being analyzed separately. Table 3 shows the yield and loading capacity of the four reaction conditions performed. It is possible to see that the lower the molar proportion between vitexin and other reagents and the shorter the reaction time, the lower the yield of the system obtained. As you increase not only the molar proportion between vitexin and the other reagents, but also the reaction time, the yield increases, as can be seen for condition number 4 (29.78% yield), which occurred with the ratio between reagents at a high level (1:10:20) and reaction time also at a high level (24 h). Figure 1A presents the response surface graph for the reaction yield and from its analysis, it is possible to observe in the darker points (in red) the conditions where it is possible to obtain the best yields for the reactions (molar ratio of vitexin:Zn:2-MeIM at 1:10:20 for 24 h).

Regarding the system loading capacity (Table 3), it is possible to identify a pattern very similar to that observed in the previous dependent variable. The lowest loading capacity (1.8 ± 0.01%) was observed in the reaction condition with both low levels (molar ratio of vitexin:Zn:2-MeIM at 0.5:10:20 for 12 h), whereas the highest loading capacity (13.02 ± 0.1%) was identified in reaction condition number 4, with both levels high. It was expected that the highest loading capacity would be observed in the condition where a larger amount of vitexin is added. Figure 1B presents the response surface graph for the loading capacity of vitexin in the system, where it is possible to observe in the red points the conditions where the best percentages of incorporation of the flavonoid in ZIF-8 were obtained (high level for both independent variables).

In a patent review published [60], the authors analyzed that the loading capacity of molecules (synthetic or natural) in MOFs can be very variable, which has been a limitation of the use of these materials in the transport of substances. It is possible to better verify this statement when analyzing recent works published with a similar theme, such as the work Bim-Júnior et al. [61] for the delivery of the flavonoid quercetin in ZIF-8, with a loading capacity of 10.4%. However, by improving the possibility of transporting natural substances in organometallic networks, a loading capacity almost three times higher (28.1 ± 2.6%) was observed by Liédana et al. [33] by encapsulating caffeine in the same MOF. Even better results can be seen in the publication by Tiwari et al. [62], who sought to encapsulate curcumin in ZIF-8 and obtained a loading capacity of 83.33%. These data prove that molecule loading can be quite variable in MOFs, especially when analyzing data related to ZIF-8. Apparently, the molecular size of the substances interferes with the loading capacity, since caffeine and curcumin are relatively smaller molecules than vitexin. Furthermore, the molecular structure seems to make curcumin fit better into the ZIF-8 pore (11.15Å) [63], since it does not have a bicyclic structure, unlike caffeine and vitexin, which have a bicyclic structure.

### 3.2. Characterization Tests

#### 3.2.1. FT-IR Analysis

**Figure 2:**
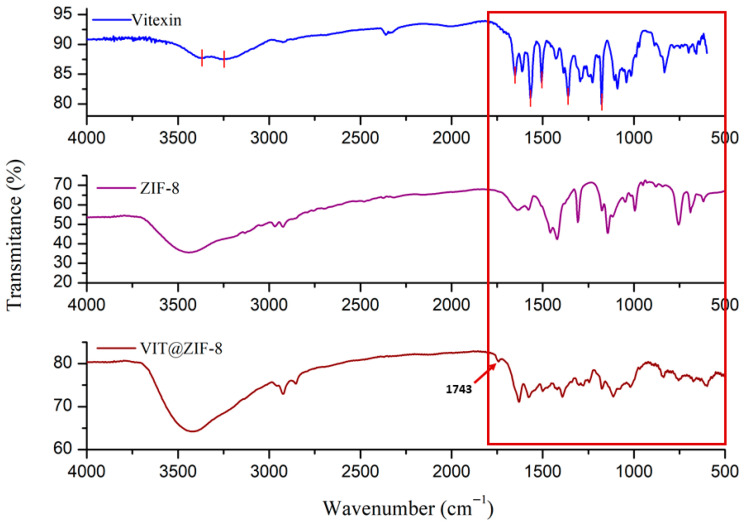
FT-IR spectra for vitexin, ZIF-8, and VIT@ZIF-8 system.

Figure 2 shows FT-IR spectra for vitexin, ZIF-8, and the system synthesized under the conditions that provided the best yield and best loading capacity. It is possible to see that the signals that stood out were those of ZIF-8, which was expected, considering that the loading of vitexin in ZIF-8 was approximately 13%; thus, the synthesized compound has a greater amount of ZIF-8 than vitexin. Still in Figure 2, the portions where the greatest differences between the FT-IR spectra were observed are highlighted in red. Bands present between 3500 and 3100 cm^−1^ (not highlighted in Figure 2) are characteristic of stretching of primary and secondary amines, as is the case of the amine present in the ZIF-8 structure. Absorption bands in this same absorption area in the spectrum (between 3400 and 3200 cm^−1^) are characteristic of hydroxyls with hydrogen bonds, as is the case of the hydroxyl present in position 5 of vitexin, which forms an intramolecular hydrogen bond with the carbonyl present in position 2.

A new and discrete signal, non-existent in the FT-IR spectra of vitexin and ZIF-8, was observed at 1743 cm^−1^, and may be a sign of interaction between the chemical structures of the flavonoid and the MOF. This is a region of characteristic signals for ketones (1725–1705 cm^−1^) or for esters (1750–1730 cm^−1^) [64]. The ketone group exists in the structure of vitexin, but it is not commonly observed in its FT-IR spectrum, probably due to the intramolecular interaction that occurs between the carbonyl and the hydroxyl at position 5. As the carbonyl may be a site of interaction between the structure of vitexin and ZIF-8, it is believed that this discrete signal is an indication of the formation of a single structure, the VIT@ZIF-8 system. However, the characteristic signal for conjugation of this carbonyl with the 5-position hydroxyl was not suppressed in the spectrum of the system, which leads us to believe that there may be a remnant of vitexin not linked to MOF, as can be seen in the band present at 1631 cm^−1^ in the spectrum of the VIT@ZIF-8 system. Yet, this signal (1631 cm^−1^) may also indicate amine folding (1640–1550 cm^−1^), which is a functional group present in the ZIF-8 structure.

Other characteristic signals for vitexin are present at 1577 cm^−1^, characteristic of aromatic groups (1600–1475 cm^−1^), as well as signals between 1300 and 1000 cm^−1^, which are characteristic carbon–oxygen bond patterns of ether, present in the oxygen of position 1. One of the most intense signals in the FT-IR spectrum of vitexin, present at 1568 cm^−1^ and characteristic of the conjugated carbonyl with an intramolecular hydrogen bond, had its intensity significantly reduced in the spectrum of the system, once again showing the probable interaction between the two structures based on the flavonoid carbonyl [23].

The characteristic signals for ZIF-8 can be seen in absorption bands present at 3417 cm^−1^, characteristic for stretching of primary or secondary amines (3500–3100 cm^−1^); 1631 cm^−1^, standard for the folding of these same functional groups (1640–1550 cm^−1^); 1392 cm^−1^ which is peculiar to alkane folding (1450–1375 cm^−1^); and, finally, signals present between 1350 and 1000 cm^−1^ that are typical for amines in general [65].

#### 3.2.2. Thermal Analysis

**Figure 3:**
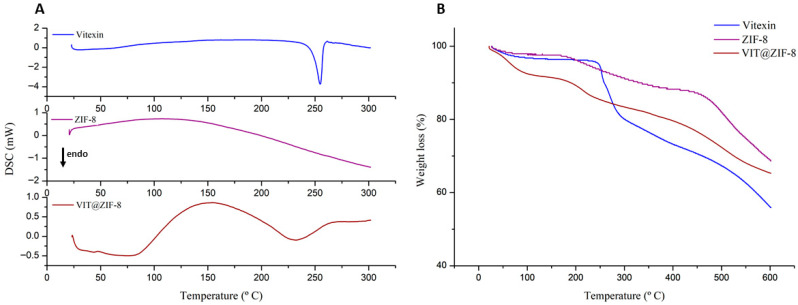
DSC and TG curves for vitexin, ZIF-8, and VIT@ZIF-8 system. Legend: (**A**)—DSC; (**B**)—TG.

Regarding analysis by differential scanning calorimetry, Figure 3A shows the DSC curves for vitexin, ZIF-8, and VIT@ZIF-8. In the DSC curve of vitexin, it is possible to observe a single thermal, endothermic event, starting at 248.06 °C and ending at 257.71 °C, representing the melting point of vitexin (T*peak* = 254.72 °C, ΔH = −212.92 mJ). In the DSC curve of ZIF-8, it is possible to notice the absence of peaks in the temperature range in which the analysis was carried out (25–300 °C), proving its thermal stability [66]. When analyzing the DSC curve for VIT@ZIF-8, it is possible to observe the absence of well-defined peaks, as well as the disappearance of the peak referring to the fusion of vitexin, which suggests the protection of the flavonoid and its incorporation into the MOF network. However, it is possible to observe an exothermic region from 85 °C onwards, and after 170 °C, a region of endothermic slope, with the absence of well-defined peaks. This type of pattern is possible and has already been reported in the literature, taking into account the type of system synthesized [67]. Therefore, it is believed that the incorporation of vitexin into ZIF-8 modified the thermal pattern of the MOF, but not enough to interfere with its thermal stability.

The TG curves are presented in Figure 3B, which show the percentage of weight loss according to the increase in temperature for vitexin, ZIF-8, and VIT@ZIF-8. Table 4 shows the thermal events present in the thermogravimetric curves for both samples. From the analysis of Table 4, it is possible to see that the thermal events evidenced are different for both samples, with the exception of the third event of ZIF-8 and VIT@ZIF-8, which presented similar temperature ranges. This similarity can be justified by two interesting points. Firstly, it is clear that the incorporation of vitexin into ZIF-8 did not determine sufficient structural changes that would corroborate the modification of the MOF’s thermal stability. Secondly, knowing that the percentage ratio in the composition of VIT@ZIF-8 is higher for ZIF-8, more influential characteristics of the MOF in thermal analysis would be expected.

Still analyzing Figure 3B and Table 4, the first thermal event of vitexin corresponded to water loss (*onset* at 15.98 °C, *endset* at 119.04 °C and 0.5% weight loss); the second thermal event showed melting (*onset* at 249.12 °C, *endset* at 257.58 °C and 6.57% weight loss), but as a probable beginning of the thermal degradation process due to the observed weight loss; and the third event demonstrated, now in fact, thermal degradation (*onset* at 264.13 °C, *endset* at 283.27 °C and 9.43% weight loss). In the ZIF-8 TG curve, the first thermal event basically represents the loss of water, which may be contained in the pores of the MOF or on its surface (*onset* at 33.79 °C, *endset* at 72.00 °C, and 1.34% weight loss) [68]. Its second thermal event presents *onset* at 181.69 °C, *endset* at 320.33 °C, and also refers to the loss of adsorbed molecules, such as residual water or unbound 2-MeIM molecules that degrade in this temperature range [69]. Finally, the last relevant and characteristic thermal event for TG analysis of ZIF-8 is related to degradation between 445.47 °C (*onset*) and 554.79 °C (*endset*), with a weight loss of 16.69%. This thermal stability, around 400 and 500 °C, is very characteristic of this type of material and has already been demonstrated in many studies [70].

Continuing with the analysis, it can be seen that the thermal events in the TG curves of VIT@ZIF-8 are different from those obtained for vitexin and ZIF-8. This fact makes it more evident that the system was actually formed. The first thermal event of VIT@ZIF-8 has an *onset* at 43.04 °C, an *endset* at 85.04 °C, and a weight loss of 6.37%, a characteristic of the loss of water adsorbed to the system in the process of obtaining. In the second thermal event, an *onset* at 193.53 °C, *endset* at 226.81 °C, and weight loss of 4.68% are observed. This event is compatible with the degradation of 2-MeIM molecules that would possibly not be linked to the MOF structure. The last thermal event evidenced in the analysis concerns the likely beginning of degradation of the VIT@ZIF-8 system, with *onset* at 419.27 °C, *endset* at 550.56 °C, and 10.77% of weight loss.

From the above, it is possible to infer that the system provided thermal protection to vitexin, as its degradation occurs in a temperature range (after 400 °C) higher than the degradation temperature of vitexin (around 260 °C). It is also possible to notice from the observation of Figure 3B that, at the end of the analysis (600 °C), vitexin is the one that degraded the most and lost weight (43.89% loss), while ZIF-8 is the one that degraded the least and lost weight (31.38% loss). Providing further evidence that the system was formed, the TG curve for the VIT@ZIF-8 system presents an intermediate weight loss (34.71% loss).

Thermal events observed in this work are characteristic of systems developed based on MOF, such as ZIF-8, that is, new materials with good thermal stability presenting a degradation temperature higher than that of the compound studied alone. This fact can be observed in the work of Sampaio et al. [36], in which the incorporation of scopoletin into the ZIF-8 network was evidenced, thereby increasing its thermal stability from 182 °C to a temperature above 440 °C. It is also interesting to highlight that DSC data corroborate the TG data, bringing to the light of science unprecedented data on thermal analyses of a system developed based on vitexin and ZIF-8.

#### 3.2.3. SEM Analysis

**Figure 4:**
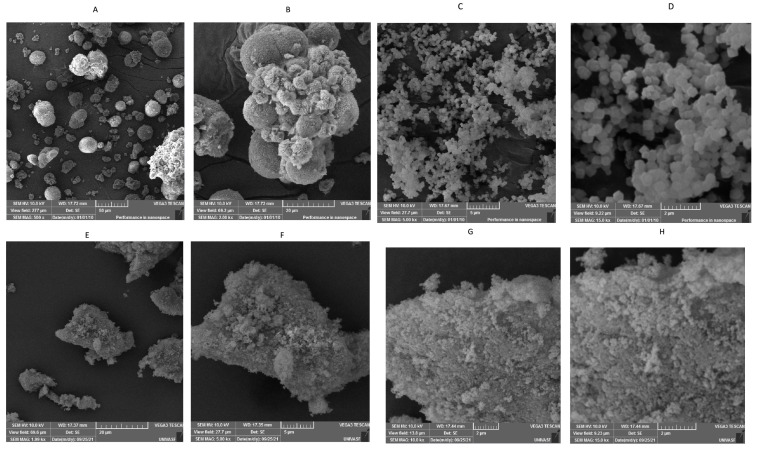
SEM photomicrographs for vitexin, ZIF-8, and VIT@ZIF-8 system. Legend: (**A**)—Vitexin (500× magnification); (**B**)—Vitexin (magnification of 2000×); (**C**)—ZIF-8 (magnification of 5000×); (**D**)—ZIF-8 (magnification of 15,000×); (**E**)—VIT@ZIF-8 (magnification of 2000×); (**F**)—VIT@ZIF-8 (magnification of 5000×); (**G**)—VIT@ZIF-8 (magnification of 10,000×); (**H**)—VIT@ZIF-8 (magnification of 15,000×).

Microscopic images of the structures of vitexin, ZIF-8, and VIT@ZIF-8 can be seen in the photomicrographs in Figure 4. SEM analysis of ZIF-8 presents uniform, small, and well-crystalline particles, with typical dodecahedral morphology [71,72] and average size of less than 1 µm (average diameter of 0.85 µm). The size range (minimum–maximum, µm) was 0.04–6.91 µm, with a median of 0.12 µm and a standard deviation of 1.78 µm. This profile is ideal for encapsulation and molecular transport, compatible with controlled release systems. The apparent uniformity and low standard deviation reinforce the quality of the synthesis and the morphological control obtained. The small dispersion and crystallinity suggest excellent thermal and structural stability, as discussed in the thermal and XRD analyses (below). The isolated vitexin presents an intermediate size between ZIF-8 and the synthesized system, with particles predominantly in the range of 3–10 µm (2.79–51.09 µm precisely, with a median of 5.88 µm and a standard deviation of 9.20 µm), but with some larger aggregates (~50 µm). The high standard deviation reinforces the intrinsic variability of natural compound particles. The morphology is irregular and lamellar, as expected from crystallized glycosylated flavonoids. Despite the heterogeneity, most of the particles have a spherical shape, as observed by Costa et al. [23].

The developed system presents heterogeneous sizes and bulky aggregates, with a maximum diameter close to 90 µm (mean diameter of 12.34 µm, with median of 1.67 µm, standard deviation of 28.86 µm, and size range of 0.74–88.97 µm). The high mean and much lower median indicate an asymmetric distribution, with few agglomerates dominating the size variation. The presence of submicrometric particles (inherited from ZIF-8) coexisting with larger agglomerates (resulting from vitexin functionalization) indicates a mixture of morphological populations. This pattern is common in functionalized or hybrid systems, where the adsorption or encapsulation of bioactive compounds can induce reorganization or growth of supramolecular domains. Regarding the morphology of the particles of the VIT@ZIF-8 system, it is not possible to perceive specific shapes, characteristics, or a defined aspect. Irregular shapes (at lower magnifications) and porous shapes (at higher magnifications) are visualized, clearly showing the amorphization of vitexin and ZIF-8 crystals.

The morphological alterations observed in the VIT@ZIF-8 system are consistent with previous reports of polyphenols encapsulation into ZIF-8, although with distinctive features that reinforce the originality of our findings. For example, quercetin-loaded ZIF-8 nanoparticles typically maintain the classical rhombic dodecahedral morphology of pristine ZIF-8, with only minor size variations and preserved crystallinity, as shown in Alginate@Quercetin@ZIF-8 nanocomposites and Qu@ZIF-8 systems designed for anti-inflammatory and chondroprotective applications [73,74]. Similarly, curcumin-loaded ZIF-8 formulations have been reported to retain well-defined crystalline structures with relatively uniform particle sizes, despite high loading capacities and pH-responsive release behavior [75,76]. In contrast, our SEM analysis revealed a heterogeneous morphology with amorphous features and larger aggregates, markedly different from both isolated vitexin and ZIF-8. Such amorphization, not previously described for flavonoid@ZIF-8 systems, is in line with XRD results and may directly contribute to the enhanced dissolution profile observed in vitro. These findings suggest that the bulkier glycosylated structure of vitexin interacts with the ZIF-8 framework in a unique manner, inducing structural reorganization that could explain its fast-release behavior and potential pharmacological performance.

#### 3.2.4. XDR Analysis

**Figure 5:**
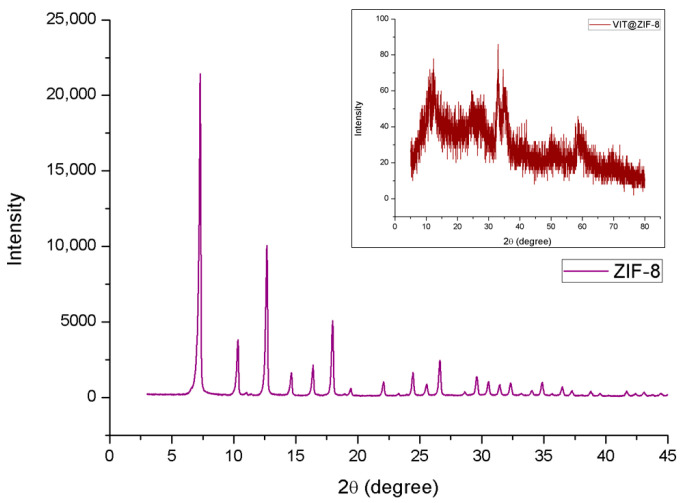
XDR patterns for ZIF-8 and VIT@ZIF-8 system.

XRD analysis was used to verify the crystallinity pattern of ZIF-8 and the system developed under the same conditions. The ZIF-8 diffractogram is shown in Figure 5, and demonstrates the highly crystalline characteristic of these structures, due to the presence of a very intense peak, such as the one at 2θ equal to 7.29° and others of medium intensity at 10.34° and 12.66°. These data corroborate with other studies that obtained similar crystallinity profiles [77,78,79].

Still in Figure 5, there is also the diffractometric profile of the VIT@ZIF-8 system, in which it was not possible to observe defined diffraction peaks (reflections) and therefore it can be inferred that the system presents an amorphous profile. This data demonstrates a new pattern of incorporation of vitexin into the ZIF-8 network, presumably resulting in an amorphous solid dispersion, providing a probable advantage in relation to the flavonoid’s pharmacological activity profiles, increasing its dissolution [80]. Another relevant point is that XRD of ZIF-8 and VIT@ZIF-8 corroborate the data obtained from SEM. Interestingly, although the XRD data revealed loss of crystallinity, the SEM micrographs (Figure 4G,H) showed that the porous morphology of the material was partially preserved. This apparent discrepancy can be explained by reports in the literature demonstrating that amorphous ZIFs may still retain irregular porous structures. For instance, Hu et al. [81] described amorphous ZIFs with disordered morphologies and preserved porosity, including core–shell architectures where a crystalline core is coated by an amorphous shell. These findings support the possibility that, even in the absence of a crystalline pattern detectable by XRD, the material may still exhibit a porous morphology visible by SEM.

### 3.3. In Vitro Release

**Figure 6:**
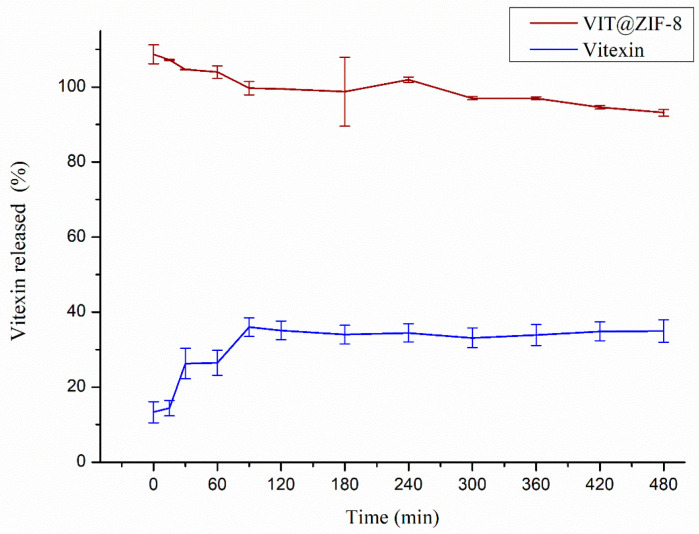
Release of vitexin and the VIT@ZIF-8 system.

The release profiles of vitexin and the VIT@ZIF-8 system in phosphate buffer (pH 7.2) are shown in Figure 6. The difference between the two release profiles is clear. After 8 h (480 min) of experiment, vitexin release was 34.98 ± 3.50%, reaffirming its low aqueous solubility or very slow dissolution rate. Unlike what happened with VIT@ZIF-8, which was able to release all of the incorporated vitexin (100%) in the first minute, thus characterizing itself as an immediate release system, positively modifying the dissolution rate of vitexin.

Solubility is one of the essential parameters measured in the pre-formulation phase, considering the fact that for any active molecule to be absorbed, it must first be solubilized in the medium, or be delivered already solubilized in an appropriate vehicle [67]. As can be seen in Figure 6, the dissolution rate of vitexin released from the system improved by 2.86 times after 8 h of experiment. This increase is even greater when observing the difference in the first minute of experimentation, in which an improvement of 7.51 times in the release of vitexin can be seen when incorporated into ZIF-8. It is expected that increasing the dissolution rate of vitexin may increase the availability of this compound in future pharmacological trials. Furthermore, the results obtained with the VIT@ZIF-8 system, indicating a complete release within 1 min, suggest significant potential for the development of a fast-acting treatment for dysmenorrhea, which could transform the clinical management of this common and often debilitating condition.

The improved dissolution profile observed for VIT@ZIF-8, which achieved complete vitexin release within 1 min, is consistent with previous reports showing that MOF-based encapsulation can markedly enhance the solubility and release of poorly soluble polyphenols. For example, hesperidin@ZIF-8 displayed a rapid and pH-dependent release under acidic conditions, directly linked to framework degradation and enhanced aqueous solubility [82]. Similarly, luteolin incorporated into β-cyclodextrin-MOFs demonstrated significantly higher dissolution rates compared to the free compound, due to improved wettability and molecular dispersion within the porous host [83]. Curcumin has also shown improved release when loaded into functionalized β-CD MOFs, with reduced crystallinity and enhanced dissolution performance compared to the free drug [84]. Taken together, these studies reinforce that the rapid and complete dissolution achieved with VIT@ZIF-8 is not a trivial outcome, but rather a direct consequence of structural amorphization and the high surface area of ZIF-8, which collectively promote immediate drug availability.

A range of approaches to improve drug solubility are used, such as the use of amorphous solid dispersions, cocrystallization, solubilization in cosolvents, use of cyclodextrins, among other forms [80,85,86]. Although these approaches can improve performance over isolated drugs, each has several disadvantages, mainly related to the chemical stability of the test substance and physical stability against crystallization. Therefore, there is still a need for methodologies to improve the solubility of the drug with the ultimate objective of stabilizing it during storage and administration [87].

MOFs can act as drug carriers, inhibiting the crystallization of these substances because they are confined within their pores, generally on a nanoscale, or through an interaction with the compound molecules on the surface of the MOF. However, they can still undergo rapid decomposition in a simulated gastric environment, leading to the immediate release of the substance, which quickly solubilizes [87]. It is believed that something similar to this may have happened with vitexin incorporated into the ZIF-8 network, which generated a significant increase in its dissolution profile. Furthermore, this data corroborates the data obtained by SEM and XRD, where it was possible to visualize the amorphization of the synthesized compound, justifying such a significant increase in dissolution rate.

### 3.4. Antidysmenorrheic Activity

Dysmenorrhea refers to menstruation accompanied by pain, and can be classified as primary (without organic pelvic lesions) and secondary (accompanied by organic lesions). Increased production of prostaglandins has been reported as one of the main causes of primary dysmenorrhea (PD), which induces uterine contractions in the endometrium [88], appearing in more than 50% of women of reproductive age [89]. Although abnormal prostanoid levels have been proposed as the main contributors to the experience of pain during PD, the etiology of this pathology remains to be fully elucidated [90].

**Figure 7:**
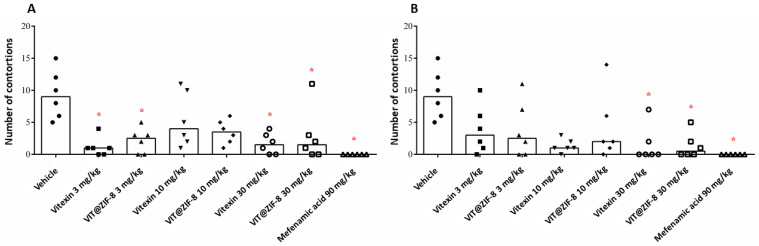
Oxytocin-induced writhing response after administration of vitexin and VIT@ZIF-8. Legend: (**A**)—orally; (**B**)—intraperitoneally.

Figure 7A shows the number of contortions that occurred after the administration of oxytocin for each sample tested (vitexin and VIT@ZIF-8 at concentrations of 3, 10, and 30 mg/kg and mefenamic acid at 90 mg/kg) orally, over a period of 30 min, compared to the vehicle (phosphate buffer). When analyzing the effects of vitexin orally, it is possible to notice that at 3 and 30 mg/kg, there is a reduction in the number of contortions when compared to the negative control (*p* = 0.0003). The same did not occur for the 10 mg/kg dose, where it was evident that there was no statistical difference when compared with the vehicle. A similar pattern of response could be observed for VIT@ZIF-8, where a reduction in the number of contortions was noticed only at doses of 3 and 30 mg/kg (*p* = 0.0008). When the vitexin and VIT@ZIF-8 groups are compared at the same doses, it is noted that there is also no difference between the isolated vitexin and that carried in the ZIF-8 network, which leads us to believe that the increase in dissolution profile of vitexin did not influence its pharmacological activity in this model.

Figure 7B shows the number of contortions that occurred for each sample tested intraperitoneally compared to the vehicle. When analyzing the effects of intraperitoneal vitexin, it is possible to notice that at doses of 3 and 10 mg/kg, no significant difference was observed when compared to the negative control (*p* = 0.0009). Unlike what was observed at a dose of 30 mg/kg, where a reduction in the number of contortions was noted. An analogous pattern of response could be observed for VIT@ZIF-8, where a decrease in the number of contortions was noticed only at the highest dose tested (30 mg/kg). When the vitexin and VIT@ZIF-8 groups intraperitoneally are checked at the same doses, it is noted that there is also no difference between the isolated flavonoid and the synthesized system, a pattern similar to the response observed for its oral administration.

These findings align with a growing body of evidence showing that specific flavonoids exert antidysmenorrheic-like effects by dampening prostaglandin-driven and oxytocin-induced uterine contractility. Quercetin, for example, inhibits PGF_2_α-induced uterine contractions in vitro and in vivo and reduces spontaneous/agonist-induced myometrial activity, supporting a direct uterine relaxant action [91]. Beyond quercetin, apigenin, naringenin, and luteolin suppress pro-labour mediators (e.g., COX-2/NF-κB pathways, cytokines) in human gestational tissues, a mechanism consistent with reduced prostaglandin signaling and contractility [92]. Flavones from *Scutellaria baicalensis* (wogonin and oroxylin A) also show robust tocolytic activity, relaxing rat uterus and inhibiting contractions triggered by oxytocin, PGF_2_α, and acetylcholine, indicating convergent modulation of Ca^2+^-dependent excitation–contraction coupling [93]. Together, these reports position vitexin within a pharmacophore class whose members attenuate uterine hyperreactivity via complementary anti-inflammatory and ion-handling effects, reinforcing the translational plausibility of the antinociceptive benefits observed in our oxytocin-induced PD model.

Oxytocin is a peptide hormone that stimulates uterine contractions through functional coupling to the oxytocin receptor (OTR). This receptor is overexpressed when activated by estrogen hormones, such as estradiol, administered in pre-treatment, and OTR concentrations determine the sensitivity of the endometrium to oxytocin stimulation. Therefore, the greater the expression of these receptors, the greater the chance of uterine contractions occurring in the presence of oxytocin [94,95].

The OTR receptor is functionally coupled to G_q/11_ protein in myometrial cells and is involved in activation of the phospholipase C enzyme, forming second messengers. Once activated, phospholipase C degrades phosphatidyl inositol-4,5-bisphosphate (PIP_2_) present in the cell membrane into inositol 1,4,5-triphosphate (IP3) and 1,2-diacylglycerol (DAG). IP3, given its water-soluble structure, migrates through the cytosol and binds to specific IP3 receptors in the endoplasmic reticulum and mitochondria, promoting the release of Ca^2+^ ions into the cytosol and increasing the concentration of this ion abruptly. DAG is associated with the plasma membrane due to its hydrophobic structure, having the function of activating protein kinase C (PKC), an enzyme linked to the plasma membrane that promotes the phosphorylation of radicals in several intracellular proteins, which culminates in an increase in the concentration of intracellular calcium. Ca^2+^ functions as a new messenger that is capable of triggering intracellular responses, such as muscle contractions [96,97,98].

One of the causes that has been most reported for PD is the increased levels of prostaglandins PGF_2α_ and PGE2, which cause myometrial contractions, ischemia, and sensitization of nerve endings, which increases the painful sensation. These chemical mediators are synthesized through the action of the enzyme cyclooxygenase 2 (COX-2) on arachidonic acid [18]. Furthermore, the role of aldose reductases in the conversion of PGH_2_ to PGF_2α_ is now fully recognized. Women with more severe dysmenorrhea have similarly elevated levels of PGF_2α_ in their menstrual fluid. These levels are highest during the first two days of menstruation, when symptoms increase. Another factor that has been closely related to pain is reduced uterine blood flow, possibly caused by strong and abnormal contractions, which results in myometrial ischemia and produces cramping [41,99].

### 3.5. Evidence of the Mechanism of Action by Molecular Docking and Dynamics

Considering the experimental pharmacological data involving vitexin as well as the relationship between PGF_2α_ synthesis and dysmenorrhea, in silico studies were performed to demonstrate the possible mechanisms of action of this flavonoid. As explained in the methods, the structures of AKR1C3 and COX-2 obtained by crystallography and properly complexed with the experimental pharmacological standard used in this study (mefenamic acid) were used for comparison. In addition, the availability of crystallographic AKR1C3 with a generic flavonoid allowed the generation of more useful data.

An important aspect to highlight is the strategic choice of combining an in vivo pharmacological model based on oxytocin-induced uterine contractions with in silico molecular modeling focused on AKR1C3 and COX-2 enzymes. The oxytocin-induced writhing model was selected because it closely mimics the enhanced uterine contractility observed in primary dysmenorrhea (PD), where increased estrogen levels lead to upregulation of oxytocin receptors and heightened myometrial sensitivity to contractile stimuli. Moreover, oxytocin-induced uterine responses are known to be modulated by prostaglandins, especially PGF_2_α, which is elevated during PD and contributes to both pain and abnormal contractility. This model allowed us to evaluate the functional effect of vitexin on uterine hyperreactivity under pathophysiologically relevant conditions.

Simultaneously, the in silico molecular docking and dynamics studies were designed to explore potential molecular targets involved in prostaglandin biosynthesis, focusing on AKR1C3 and COX-2, both of which play critical roles in PGF_2_α production. This computational approach aimed to provide mechanistic insights at the molecular level, complementing the in vivo findings.

Although we recognize the value of direct biochemical assays—such as in vitro enzyme inhibition or PGF_2_α quantification in uterine tissue—to confirm the interaction of vitexin with these targets, these experiments were beyond the scope and available resources of this initial study. Nevertheless, the combination of functional in vivo results and target-based computational evidence provides a rational foundation for future mechanistic studies, which will include enzymatic activity assays and uterine prostaglandin profiling to validate the predicted molecular interactions.

The redocking results for the three crystallographic complexes allowed a better definition of the grid box dimensions. The RMSD results for all crystallographic complexes and their respective interaction energy with the vitexin ligand can be seen in Table 5. Evaluating the data in this table, it can be observed that Autodock Vina was able to reach all crystallographic geometries (RMSD < 2 Å). The docking results indicated that, within the AKR1C3 active site, vitexin and mefenamic acid displayed nearly equivalent binding affinity scores (ΔE = 0.2 kcal/mol). Moreover, when vitexin was compared against the crystallographic flavonoid ligand from the 8JP1 structure, it demonstrated superior stability within the binding pocket. This interaction pattern reinforces the functional connection between aldose reductase modulation, prostaglandin biosynthesis associated with dysmenorrhea, and the pharmacological evidence obtained in our experimental framework.

On the other hand, considering now the second enzyme possibly involved (COX-2), the data in Table 5 allow us to observe that mefenamic acid does behave as a good ligand for COX-2, which was expected, but vitexin did not show a satisfactory interaction energy, with a stabilization much lower than the standard drug. These findings, in turn, point to vitexin having a more selective action in dysmenorrhea at the level of aldose reductase, disfavoring the attribution of the COX-2 pathway.

Seeking to bring a more robust content to the previous in silico analyses, molecular dynamics simulations were performed on the crystallographic complexes, with their respective ligands, as well as after docking with vitexin. It is important to add that, in the case of the protein conformation in AKR1C3 corresponding to 8JP1, vitexin presented two important results: the most stable docking pose had a spatial arrangement distant from the crystallographic ligand, an inevitable comparison made because the latter is a flavonoid. However, the sixth most stable pose of vitexin had a geometry compatible with the standard ligand. Thus, we called the first docking result of vitexin “vitexin best energy” (VBE) and the geometry closest to crystallographic, although less stable, “vitexin best geometry” (VBG). These precautions were taken to gather the largest possible amount of data on these systems.

**Figure 8:**
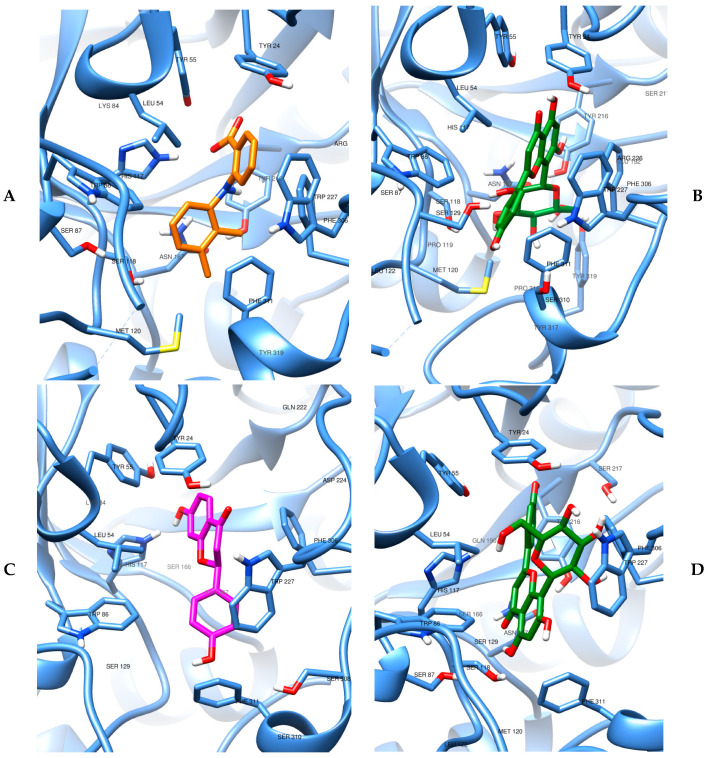

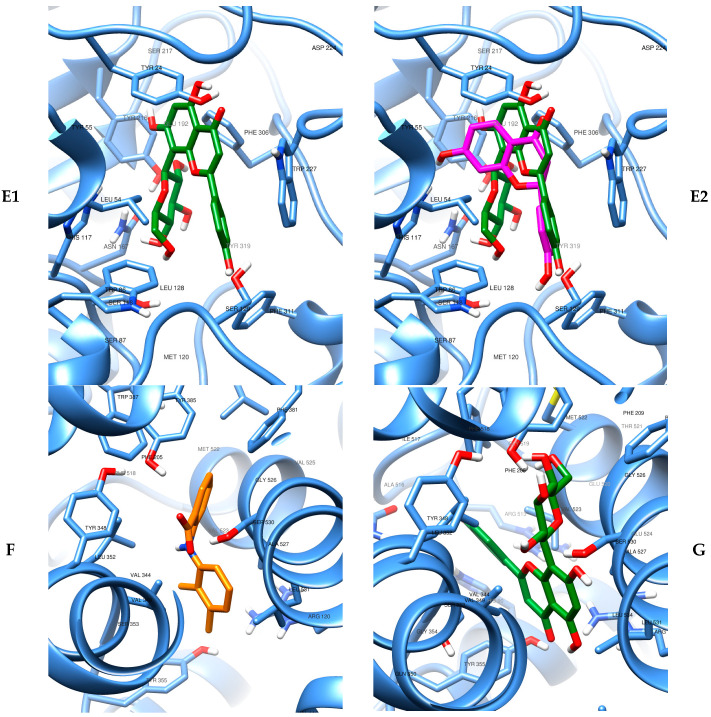
Complexes resulting from molecular docking calculations used as input for molecular dynamics simulations. Legend: (**A**,**B**)—AKR1C3 interacting with mefenamic acid (orange) and vitexin (green), respectively; (**C**,**D**)—AKR1C3 interacting with crystallographic flavonoid (magenta) and vitexin (in VBE format), respectively; (**E1**,**E2**)—AKR1C3 interacting with vitexin (in VBG format) and this overlapping this with crystallographic flavonoid, respectively; (**F**,**G**)—COX-2 interacting with mefenamic acid and vitexin, respectively.

The images of molecular docking results used as a starting point for the molecular dynamics simulations can be visualized in Figure 8. We can observe that the catalytic site of both enzymes is rich in amino acids with nucleophilic potential, especially the common presence of the amino acid serine, in addition to aromatic systems capable of stabilizing via stacking interactions. In this sense, it is plausible to observe that the rich presence of hydroxyls and other oxygenated functions, as well as ring systems of the vitexin glycoside, facilitates the achievement of stable interactions. The more open binding site in AKR1C3, combined with the presence of more amino acids of a conjugated cyclic nature (tryptophan, histidine, tyrosine, phenylalanine), justifies a more satisfactory affinity in comparison to COX-2 for vitexin.

Detailing a little more the structural characteristics of each binding site, the target AKR1C3 in 3R43 has 323 aa. In the complex, mefenamic acid occupies the enzyme pocket in a manner consistent with NSAID binding across sub-pockets 1 and 2–SP1/SP2 and the Steroidal Channel–SC: its carboxylate can participate in H-bonding within the Tyr55/His117/NADP^+^ oxyanion region, while the two aromatic rings form extensive hydrophobic/π–π interactions (commonly involving Trp227 and phenylalanine residues along SP1/SP2). The favorable docking profile of vitexin in the AKR1C3 binding site has important structural and electronic justifications. The multiple hydroxyl groups in the flavonoid backbone allow for an extended hydrogen bonding network with residues of the catalytic tetrad, particularly the multiple tyrosines and His117, as well as with the SP1 subpocket, thus compensating for the absence of a carboxylate anchor. At the same time, the vitexin rings are shown to engage in π-π stacking and dispersion interactions with Trp227, Phe306, and Phe311 within the hydrophobic SP2 region, similar to some NSAIDs such as mefenamic acid. The bulky β-glucopyranosyl substituent attached to the flavone backbone can be accommodated in the relatively exposed environment of the AKR1C3 cavity, where it can also contribute hydrogen bonding. Taken together, these complementary polar and hydrophobic contacts justify the good docking affinity of vitexin for AKR1C3, highlighting the structural adaptability of the active site of this enzyme and its ability to stabilize non-classical ligands through the concerted coupling of SP1 and SP2.

AKR1C3 of the 8JP1 complex also has 323 amino acids. This enzyme captures the canonical NADP^+^-dependent catalytic tetrad (Tyr55, Asp50, Lys84, His117) and the oxyanion site formed by Tyr55/His117/NADP^+^, which supports hydride transfer. Ligands typically engage a set of sub-pockets—the steroid channel (SC) and SP1/SP2/SP3—allowing combinations of H-bonding to the Tyr55/His117/NADP^+^ triad and hydrophobic/π interactions with residues lining the pocket (often including Trp227 and phenylalanines that define SP1/SP2). The DFV complex (8JP1) exemplifies this binding mode and provides the current crystallographic context for AKR1C3 inhibition. When docked in the AKR1C3 structure of the PDB:ID 8JP1 complex, vitexin exhibits favorable complementarity with the NADP^+^-dependent catalytic site and surrounding subpockets. The VBE and VBG conformations fit comfortably within the binding site, occupying the same volume, but inverted. In the vitexin (VBG) conformation most similar to the co-crystallographic flavonoid (DFV), the sugar moiety is oriented toward the catalytic tetrad (Tyr55, Asp50, Lys84, His117), establishing several hydrogen bonds and van der Waals interactions. The condensed ring system projects toward the hydrophobic SP2 site, where it can form π-π stacking and van der Waals contacts with aromatic residues Trp227, Phe306, and Phe311, thus mimicking the binding strategy of known NSAID inhibitors. The first residue also shows a possible interaction with the phenolic group of vitexin. Considering the VBE geometry, the presence of the glycoside in vitexin, unlike the co-crystallographic ligand, opens up a new field of interactions, this time with the condensed ring system oriented toward the catalytic tetrad and the carbohydrate moiety toward Trp277, also establishing several hydrogen bonding and van der Waals contacts. Its lower energy will later be tested in relaxation molecular dynamics simulations. We then have a structural justification and conformational possibilities that denote the entropic favoring of vitexin in the favorable anchoring of another AKR1C3 complex.

Finally, the COX-2 is a homodimeric enzyme with sequence of 604 amino acids for humans. In 5IKR, mefenamic acid occupies the catalytic/entrance region, interacting with Arg120 and Tyr355 (typical H-bond/salt-bridge partners for acidic NSAIDs), beyond Tyr385 and Ser530 (with Gly526/Ala527 lining the channel). The ligand is also stabilized by hydrophobic/π contacts with Val349, Leu352, Phe381, Leu384, Trp387, Phe518, Val523; other aa, such as Arg513 and His90, can contribute polar contacts that differentiate COX-2 from COX-1. Fenamates (including mefenamic acid) characteristically bind in an inverted orientation in COX-2, positioning the carboxylate toward Arg120/Tyr355 and the diarylamine scaffold deep into the hydrophobic channel. When vitexin was docked to COX-2, the very poor binding energy relative to mefenamic acid can be rationalized by structural and electronic considerations. Unlike fenamates, vitexin lacks a carboxylate moiety capable of forming the canonical salt bridge and hydrogen bonds with Arg120 and Tyr355, which normally serve as anchor residues at the channel entrance. Furthermore, the bulky structure enhanced by the β-glucopyranosyl substituent is poorly accommodated in the predominantly hydrophobic tunnel formed by residues such as Val349, Leu352, Phe381, Leu384, Trp387, Phe518, and Val523. The sugar moiety introduces excessive polarity and steric hindrance, preventing deep penetration of the ligand into the catalytic cavity where Tyr385 and Ser530 act in substrate oxidation. Although multiple hydroxyl groups in the flavonoid can establish hydrogen bonds, these interactions are insufficient to compensate for the enthalpic and entropic penalties associated with poor geometric and chemical complementarity. Thus, vitexin cannot reproduce the productive network of electrostatic, hydrogen-bonding, and π-π/hydrophobic contacts observed with mefenamic acid, explaining its less negative (less favorable) docking energy and the predicted low affinity for COX-2.

**Figure 9:**
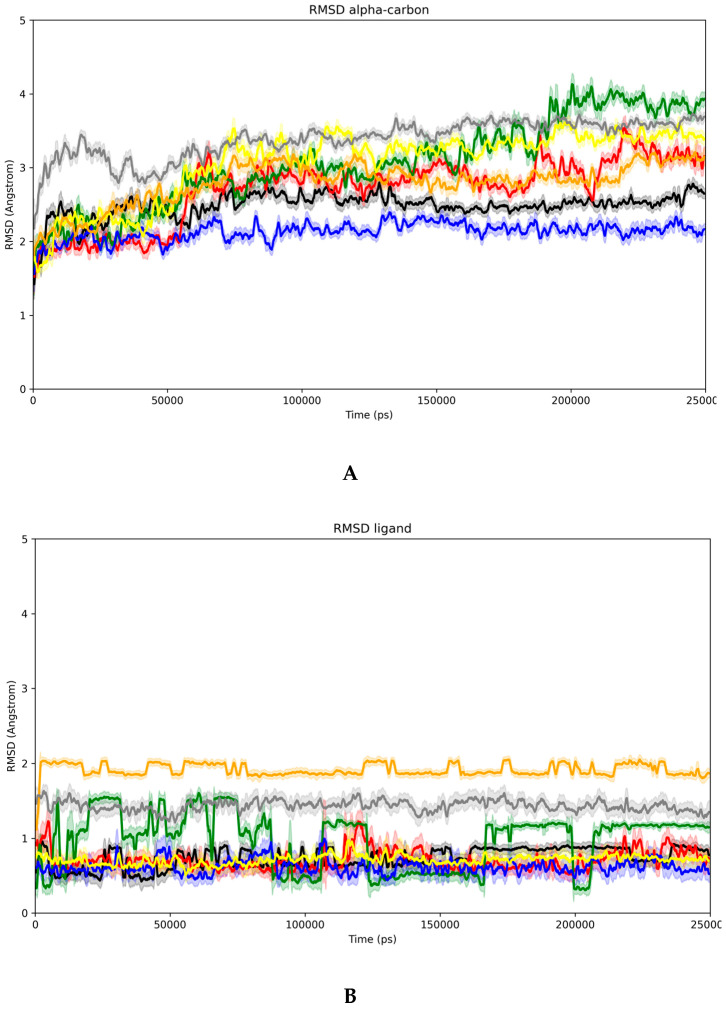

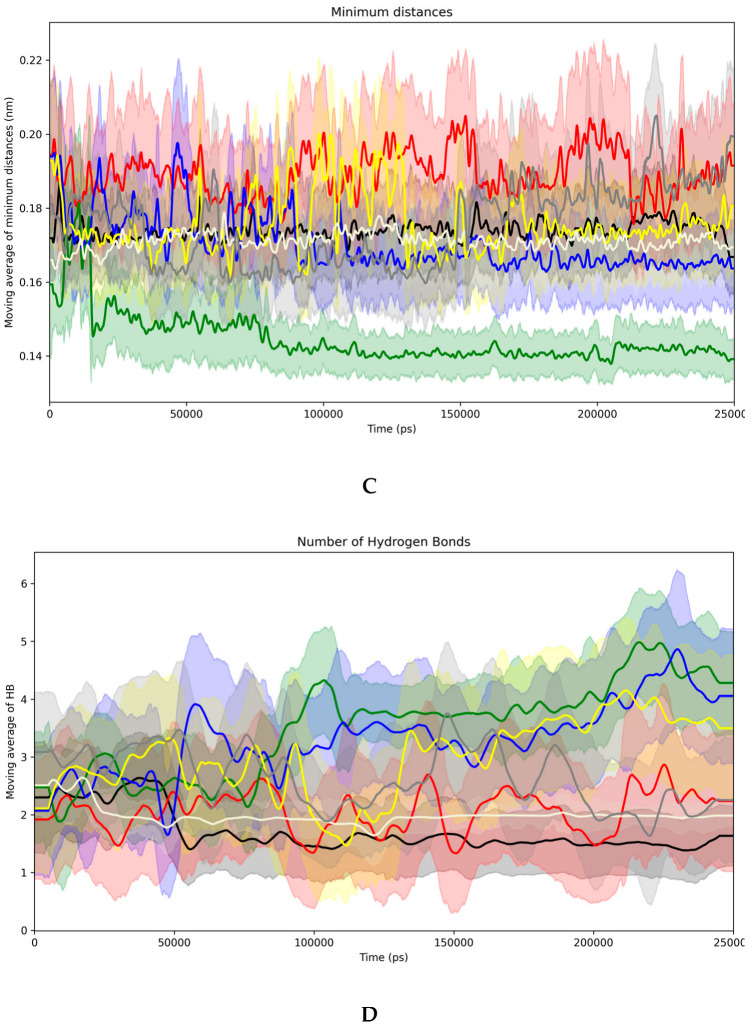
Results obtained from the molecular dynamics simulations. Legend: (**A**)—3D complex format. Three-dimensional complex format for alpha-carbons of complexes ARK1C3/MFA (Black), ARK1C3/VTX (Red), ARK1C3/DFV (Green), ARK1C3/VBE (Blue), ARK1C3/VBG (Yellow), COX-2/MFA (Orange), COX-2/VTX (Grey). (**B**)—RMSD for ligands in the complexes above. (**C**)—Minimum distance plot for ligand–protein complexes in the same color sequence as above (except for the orange color being replaced by beige) (Values were converted to a moving average every 100 ps for curve smoothing, with standard deviation shown in shading). (**D**)—Number of hydrogen bonds plot for ligand–protein complexes in the same color sequence as above (except for the orange color being replaced by beige) (Values were converted to a moving average every 500 ps for curve smoothing, with standard deviation shown in shading).

In turn, the results obtained from the molecular dynamics simulations can be seen in Figure 9 and Table 6. Initially, the analysis of the RMSD (Å) for the alpha carbons of the respective proteins along the trajectory between 20 and 250 ns allows us to evaluate the degree of general stabilization of the complexed macromolecules.

In Figure 9A, we check the moving average of RMSD every 100 ps of simulation, whose data is complemented by the respective standard deviation in shaded. This technique allows us to smooth the curve for a cleaner analysis of the trajectory. The black line corresponds to AKR1C3 complexed with mefenamic acid (MFA), and the red line when this enzyme is complexed with vitexin. Both proteins show a variation of less than 1A over the production time, with the complex with MFA leading to better stability in relation to the initial conformation and less fluctuation along the trajectory.

The green line (Figure 9A) denotes the simulation of AKR1C3 with the crystallographic flavonoid, showing significant deviations over the simulation time. This becomes more evident when we compare this same protein now with vitexin in VBE format, represented by the blue line. In fact, using the response of the best energy docking, we have a much more stable simulation regarding the alpha carbons than using the conformation with geometry close to the crystallographic flavonoid (VBG), represented by the yellow color. We then verified that vitexin forms a stable complex in AKR1C3, both starting from a protein geometry complexed with MFA and a crystallographic flavonoid, this effect being even more evident in the second case.

For the complexes with COX-2, the orange line represents the trajectory of the alpha carbons of this protein with MFA, while the gray line represents the trajectory of the alpha carbons of this protein with vitexin. There is no significant difference in the RMSD along the trajectories analyzed, although when complexed with vitexin, the protein experiences a greater global variation in relation to the starting point. This, however, may indicate accommodation and not necessarily instability.

Regarding the RMSD of the ligands (Figure 9B), we highlight only the relatively unstable behavior of the crystallographic flavonoid in AKR1C3 (green line), as well as the higher global difference between both MFA and vitexin in the COX-2 environment (orange and gray lines), facts that are in line with the observations for the RMSD of the alpha carbons.

About the minimum distances ligand–protein (Figure 9C), we verified that the co-crystallographic compounds (mefenamic acid and flavonoid) in their respective receptors have a more homogeneous behavior of minimum distances (black, green, and beige) compared to vitexin (red, blue, and gray). In all cases, no distances greater than 0.2 nm (2 Angströms) were observed. Observing the behavior of DFV at 9C (green line), the minimum distance observed between this molecule and the protein is the smallest among the complexes analyzed. It is important to clarify that this data should not be taken rigidly as a stability criterion, but rather allows us to infer that the compound remains in the binding site during the simulation, since only the sum of all other contacts a molecule makes can attest to its absolute stability.

Regarding the number of hydrogen bonds formed along the trajectory (Figure 9D), we observed that the AKR1C3 complex with mefenamic acid (black) forms fewer hydrogen bonds than when we have the presence of vitexin, which could be expected considering the greater number of possibilities with the latter. In this same type of receptor, compared to the co-crystallographic flavonoid, vitexin maintains a similar number of hydrogen bonds along the trajectory. In the case of vitexin with COX-2, in gray, we observe a greater number than with mefenamic acid (beige), as denoted by the greater number of hydroxyls in that molecule. On average, a higher absolute number of hydrogen bonds is observed for DFV (green line). However, the blue line, corresponding to VBE, is very close, even surpassing it at some points, especially if we consider the deviation from the truncation of the number of hydrogen bonds along the trajectory (shaded).

Table 6 presents the average binding energies for the stretch of the trajectory between 240 and 250 ns, chosen due to the homogeneity of all RMSD alpha plots in this region. It was possible to observe that for AKR1C3 with mefenamic acid (MFA) or vitexin (VTX), the interaction energy was practically the same, with a smaller deviation fluctuation for vitexin. The value denotes the formation of a stable complex for both molecules, with the electrostatic contribution of MFA being greater, probably due to the ionized carboxyl, while the van der Waals contribution of VTX was more prominent, probably due to the larger surface area and free hydroxyls.

From the fifth to the sixth columns of Table 6, we observed a significant stabilization of vitexin in AKR1C3 compared to the crystallographic flavonoid (FLV) in this starting conformation of the enzyme, reinforcing the excellent stability of VTX as an aldose reductase inhibitor. The last two columns of the same table show that the interaction of COX-2 with mefenamic acid is less stable than that of aldose reductase. Furthermore, vitexin showed even lower stability, much lower than the previous values for AKR1C3.

The molecular dynamics and docking data, taken together, represent strong evidence of the mechanism of action of vitexin via aldose reductase, corroborating existing data in the literature on the role of this enzyme in the synthesis of PGF_2α_ and dysmenorrhea.

It is important to highlight that the molecular docking and dynamics simulations conducted in this study were performed using the isolated structure of vitexin, without including the VIT@ZIF-8 delivery system in the computational modeling. This choice was made because the primary role of the ZIF-8 carrier is to enhance the physicochemical properties of vitexin, particularly its solubility and stability, potentially improving its bioavailability after administration. However, once released from the carrier, the pharmacological activity of vitexin is expected to depend solely on its intrinsic ability to interact with biological targets, such as AKR1C3 and COX-2.

Supporting this rationale, our in vivo results demonstrated that both free vitexin and VIT@ZIF-8 produced comparable reductions in uterine writhing at the same doses, suggesting that the delivery system did not alter the mechanism of action at the target level. Therefore, focusing the in silico studies on the free ligand (vitexin) was considered the most appropriate approach to investigate its molecular interactions with the targets involved in PGF_2_α biosynthesis and uterine contractility regulation.

Although molecular docking and molecular dynamics (MD) simulations demonstrated a stable interaction between vitexin and AKR1C3, with a favorable binding energy (−132.22 kcal/mol), we recognize that these in silico results cannot be directly extrapolated to predict the in vivo pharmacodynamic dose required for therapeutic effect. The observed pharmacological response at 30 mg/kg likely reflects not only the affinity for AKR1C3 but also the influence of multiple pharmacokinetic factors, such as absorption, distribution, metabolism, and bioavailability, all of which are particularly relevant for flavonoid compounds. Additionally, vitexin may exert its effects through multiple molecular targets, including COX-2 and pathways modulating uterine contractility, which further complicates establishing a direct correlation between docking-derived binding energies and the effective in vivo dose.

We also acknowledge that the determination of IC_50_ values for vitexin against AKR1C3 and COX-2 would provide a more quantitative and mechanistic understanding of its inhibitory potential compared to the reference drug, mefenamic acid. However, due to logistical and resource constraints, these biochemical assays were not performed in this study. Future work will prioritize these experiments, enabling a more robust comparison between computational predictions, in vitro enzyme inhibition, and in vivo pharmacodynamic outcomes.

### 3.6. In Silico ADME Profile

Table 7 below presents the ADME profile for vitexin, followed by a discussion about its structure–pharmacokinetics relationships and possibilities.

In the Table 7, although SwissADME predicted vitexin to be “soluble” (Log S between −2.38 and −3.57, corresponding to a solubility range of 0.1–1 mg/mL), the experimentally determined aqueous solubility is only 7.62 µg/mL (≈7.6 × 10^−3^ mg/mL; Log S ≈ −4.75), i.e., nearly two orders of magnitude lower than the computational estimate. This discrepancy can be rationalized by considering the physicochemical and structural features that limit dissolution in real systems but are not fully accounted for in topological or fragment-based models such as ESOL or SILICOS-IT.

Vitexin is a crystalline polyphenolic flavone-C-glycoside with multiple hydroxyl groups (seven hydrogen-bond donors) and extensive intermolecular hydrogen bonding. These features increase lattice energy and promote crystal packing stability, substantially reducing the free energy of dissolution. Moreover, the molecule is prone to self-association through π–π stacking and hydrogen-bonded aggregates in aqueous media, further decreasing the concentration of free monomers. SwissADME models, by contrast, assume an idealized monomeric state and neglect solid-state effects such as polymorphism, hydration, or aggregation.

The poor intrinsic solubility also reflects limited ionization at physiological pH, since the phenolic hydroxyl groups have pKa values generally >8. Thus, vitexin remains predominantly neutral across the gastrointestinal pH range, restricting dissolution enhancement through ionization. In practice, this means that even though in silico tools predict moderate hydrophilicity (consensus *Log(P)* ≈ 0), the compound behaves experimentally as a poorly soluble but hydrophilic crystalline solid.

Considering the exposure above, focusing now on a possible oral administration, the combination of low solubility and high topological polar surface area (*TPSA* = 181 Å^2^) leads to dissolution-limited absorption. Enhancing oral bioavailability, therefore, requires strategies that improve both solubility and permeability. In this sense, nanoencapsulation in metal–organic frameworks, particularly ZIF-8, can protect phenolic moieties, provide pH-responsive release, and improve mucosal permeation.

For intravenous administration, the intrinsic solubility of 7.6 µg/mL is still too low for direct aqueous injection without co-solvents or nanocarriers. Encapsulation in ZIF-8 or liposomal systems may allow safe parenteral delivery by increasing apparent solubility and controlling systemic release, while preventing rapid phase II conjugation (glucuronidation/sulfation).

In summary, while SwissADME predictions overestimate vitexin’s solubility due to model simplifications considering individual molecules, the experimental data confirm that it behaves as a poorly soluble polyphenolic compound. Consequently, advanced formulation strategies, as nanocarriers such as ZIF-8 frameworks, are justified to achieve therapeutic concentrations either orally or intravenously, reconciling the computational and experimental evidence within a coherent pharmacokinetic framework.

## 4. Conclusions

This study successfully developed and characterized an immediate-release system (VIT@ZIF-8) incorporating vitexin into a ZIF-8 framework through a 2^2^ factorial design. The optimized formulation demonstrated a 29.78% synthesis yield and 13.02% loading capacity, confirming efficient encapsulation of vitexin. Spectroscopic, thermal, and morphological analyses (FT-IR, DSC/TG, SEM, and XRD) revealed the formation of a new amorphous hybrid material with preserved structural stability and enhanced physicochemical compatibility between the flavonoid and the MOF network. The amorphization observed was directly associated with a remarkable improvement in dissolution behavior —achieving complete vitexin release within one minute, a 7.5-fold increase compared to the free compound, confirming the system as an immediate-release platform.

Pharmacological evaluation demonstrated that both free vitexin and the VIT@ZIF-8 formulation significantly reduced oxytocin-induced abdominal writhing at oral doses of 3 and 30 mg/kg, comparable to the positive control. Although the MOF-based system did not potentiate the in vivo effect relative to isolated vitexin, the combination of in vivo and in silico analyses provided new mechanistic insights. Molecular docking and dynamics revealed that vitexin interacts favorably with aldose reductase (AKR1C3), suggesting this enzyme as a relevant molecular target for its antidysmenorrheic activity, while showing low affinity for COX-2. These findings indicate a non-classical, selective mechanism distinct from conventional NSAIDs.

Overall, the VIT@ZIF-8 system represents a novel technological approach capable of overcoming the poor aqueous solubility of vitexin without compromising its pharmacological activity. The evidence generated supports vitexin as a promising lead compound for the development of alternative or adjuvant therapies for primary dysmenorrhea. Future studies should focus on mechanistic validation through enzymatic assays of AKR1C3 inhibition, prostaglandin quantification in uterine tissues, and evaluation of pharmacokinetic and safety profiles to advance the translational potential of this innovative platform.

## Figures and Tables

**Table 1:** Variables and levels of the experimental design for the study.

Independent Variables	Levels	
	−1	+1
Molar proportion between vitexin and reagents (Vitexin:Zn:2-MeIm)	0.5:10:20	1:10:20
Reaction time	12 h	24 h

(−1) Low level; (+1) High level.

**Table 2:** 2^2^ Factorial planning matrix.

Reaction Condition	Molar Proportion Between Vitexin and Reagents	Reaction Time
1	−1 (vit 4 μM)	−1 (12 h)
2	−1 (vit 4 μM)	+1 (24 h)
3	+1 (vit 8 μM)	−1 (12 h)
4	+1 (vit 8 μM)	+1 (24 h)

(−1) Low level; (+1) High level.

**Table 3:** Yield and loading capacity of the synthesis conditions of the VIT@ZIF-8 system.

Reaction Condition	Reaction Yield (%)	Loading Capacity (%)
1 (−1, −1)	7.33	1.80 ± 0.01
2 (−1, +1)	18.73	1.91 ± 0.01
3 (+1, −1)	26.94	4.75 ± 0.015
4 (+1, +1)	29.78	13.02 ± 0.1

**Table 4:** TG thermal events for vitexin, ZIF-8, and the VIT@ZIF-8 system.

Sample	1st Event			2nd Event			3rd Event		
	Onset (°C)	Endset (°C)	Weight loss (%)	Onset (°C)	Endset (°C)	Weight Loss (%)	Onset (°C)	Endset (°C)	Weight Loss (%)
Vitexin	15.98	119.04	0.5	249.12	257.58	6.57	264.13	283.27	9.43
ZIF-8	33.79	72.00	1.34	181.69	320.33	8.42	446.47	554.79	16.94
VIT@ZIF-8	43.04	85.04	6.37	193.53	226.81	4.68	419.27	550.56	10.77

**Table 5:** RMSD, redocking energy, and binding energy for complexes tested.

Complex	RMSD (Å)	Redocking Energy (Kcal/mol)	BE * with Vitexin (Kcal/mol)
3R43 (AKR1C3 with mefenamic acid)	0.348	−9.1	−8.9
8JP1 (AKR1C3 with a flavonoid)	0.486	−8.2	−8.6
5IKR (COX-2 with mefenamic acid)	1.848	−9.1	−5.9

* Binding energy.

**Table 6:** Results obtained from the molecular dynamics simulations.

Energy	AKR1C3/MFA	AKR1C3/VTX	AKR1C3/FLV	AKR1C3/VBE	AKR1C3/VBG	COX-2/ MFA	COX-2/ VTX
Binding energy	−131.375 ± 21.313	−132.220 ± 14.985	−42.005 ± 18.369	−111.644 ± 18.416	−95.586 ± 16.440	−49.112 ± 68.874	−18.785 ± 87.254
Van der Waals	−128.159 ± 11.871	−202.753 ± 12.809	−89.788 ± 18.554	−215.007 ± 13.086	−214.191 ± 10.119	−45.259 ± 65.358	−67.208 ± 98.803
Electrostatic	−342.275 ± 29.052	−28.896 ± 9.564	−193.187 ± 17.586	−57.177 ± 11.189	−63.589 ± 10.840	−55.516 ± 70.586	−7.582 ± 12.081
Polar solvation	354.146 ± 28.074	121.333 ± 11.011	256.122 ± 17.874	182.100 ± 15.582	204.050 ± 15.537	56.810 ± 93.724	63.874 ± 46.987
SASA	−15.087 ± 0.731	−21.904 ± 1.125	−15.152 ± 0.737	−21.560 ± 0.863	−21.856 ± 1.074	−5.148 ± 7.636	−7.870 ± 11.035

**Table 7:** Main ADME parameters predicted by SwissADME.

Category	Parameter	Obtained Value	Interpretation
Physicochemical	Molecular weight (g/mol)	432.38	Moderately high; within acceptable range, but may reduce permeability.
	TPSA (Å^2^)	181.05	Very high; TPSA > 140 indicates low intestinal and BBB permeability.
	H-bond donors	7	Exceeds Lipinski’s limit (≤5); may reduce oral absorption.
	H-bond acceptors	10	At upper limit; contributes to solubility.
	Rotatable bonds	3	Low flexibility; contributes to conformational stability.
Lipophilicity	Log P (consensus)	−0.02	Neutral/hydrophilic; high solubility but limited membrane permeability.
Solubility (Log S)	ESOL: −2.84; Ali: −3.57; SILICOS-IT: −2.38	Soluble	Good aqueous solubility.
Pharmacokinetics	GI absorption	Low	Consistent with high polarity and high TPSA.
	BBB permeation	No	Expected for polar, polyphenolic compounds.
	P-gp substrate	No	Low risk of active efflux, but overall absorption still limited.
	CYP450 inhibition	No (1A2, 2C19, 2C9, 2D6, 3A4)	Low potential for metabolic drug–drug interactions.
	Log Kp (cm/s)	−8.79	Indicates low skin permeability.
Drug-likeness	Lipinski	1 violation (H-donors > 5)	Moderate compliance with drug-like profile.
	Veber	1 violation (TPSA > 140)	Reduced oral absorption expected.
	Bioavailability score	0.55	Moderate; suggests limited bioavailability.
Medicinal Chemistry	PAINS / Brenk alerts	0	No structural alerts for assay interference.
	Synthetic accessibility	5.12	Moderately complex synthesis.

## Data Availability

Data are contained within the article.

## Supplementary Materials

The following supporting information can be downloaded at: https://www.mdpi.com/article/10.3390/biomedicines13112602/s1, Table S1: Chemical shifts (δ) for vitexin. The supplementary material contains information on the isolation and structural identification of vitexin from the species *Jatropha mutabilis*, in addition to the methodology for quantifying vitexin by HPLC-DAD.
