# Supplementary material for: Vitexin as a Potential Antidysmenorrheic Agent: Development of a ZIF-8-Based Immediate-Release System and Evaluation via In Vivo and In Silico Approaches
Source: Biomedicines. 2025 Oct 24;13(11):2602. doi: 10.3390/biomedicines13112602 (PMC12650589; doi:10.3390/biomedicines13112602)
Supplement: Supplementary file 1 [file biomedicines-13-02602-s001.zip › biomedicines-3900005-supplementary.pdf]

Supplementary material

Materials and Methods

Vitexin isolation

Vitexin was obtained from the species *Jatropha mutabilis* (Pohl.) Baill (SISGEN registration A94E671). The leaves were collected (5.95 kg) at the Center for Recovery of Degraded Areas of Caatinga at Universidade Federal do Vale do São Francisco (CRAD/UNIVASF) in the municipality of Petrolina, Permabuco State, Brazil (-9º19'32.2 S 40º32'48.2 W), in March 2019. The leaves were stored in paper bags, dried in an oven with circulating air at 45º C for 72 h (1.315 kg), pulverized in a knife mill (model SL-31, SOLAB) and then macerated at room temperature with 95% ethanol. Three extractions were performed with 72 h intervals between each extraction. The extractive solution was filtered and finally concentrated in a rotary evaporator (model SL-125, SOLAB) under reduced pressure at a temperature of approximately 50°C, until the crude ethanolic extract (CEE) was obtained (264.1g, 4.43% yield from fresh leaves) [1].

The CEE (200 g) was solubilized in a mixture of water and methanol (7:3), mechanically stirred for 1 h and partitioned with hexane, chloroform and ethyl acetate, generating three fractions of distinct polarities. The fraction of interest (ethyl acetate) was concentrated in a rotary evaporator (50° C) at reduced pressure, until a precipitate was obtained (later identified as vitexin). After that, the precipitate was washed with methanol several times to remove possible impurities until a light yellow powder was obtained (950 mg, 0.457% yield from CEE and 0.09% yield from dry leaves). This light yellow powder was analyzed by Nuclear Magnetic Resonance (NMR). NMR experiments were performed on a Bruker AscendTM 400 instrument, operating at 400 MHz for ^{1}H NMR and at 100 MHz for ^{13}C. The samples were solubilized in deuterated dimethyl sulfoxide (DMSO-d_6) (Tedia®, Brazil). Chemical shifts were expressed in ppm (δ)[2].

Vitexin quantification by HPLC-DAD

To determine the loading capacity, 5 mg of VIT@ZIF-8 was dispersed in 10 ml of distilled water and acidified with drops of trifluoroacetic acid until pH 1-2, where the ZIF-8 network is broken, releasing vitexin. The solutions were filtered through a membrane with a pore size of 0.45 μm (Chromfilter®) and analyzed in a high performance liquid chromatograph coupled to a diode array detector (HPLC-DAD, model LC-10ADVP, Shimadzu®), with thermostated column compartment (model CTO, 10ASVP), automatic injector (model SIL, 20ADVP), controller (model SCL, 20ADVP) and degasser (model DGU, 20A). Data collection and analyzes were performed using Shimadzu® LCSolution™ software. An RP-18 column (150 x 4.6 mm), particle size 2.7 μm (Poroshel, Agilent®) was used. The mobile phase was composed of acetonitrile as solvent A and 0.1% formic acid in ultrapure water as solvent B. Separations were carried out by a gradient as follows: 0-40 min, 90-72% solvent B; 40-50 min, 72-90% solvent B; 50-60 min isocratic. The mobile phase flow rate was 0.4 ml/min, with an injection volume of 10 μl. All chromatographic tests were carried out at 37º C, with UV detection at 336 nm, in triplicate. The areas of the chromatographic peaks were used as a response to the concentration of the solution used, calculated by the equation of the straight line obtained from the calibration curve ($y = 93716.61x – 25795.94$) [1].

Results and Discussion

Isolation and structural identification of vitexin

Vitexin is not a new substance, and its isolation and structural identification have been reported in the literature several times, which facilitates the determination and assignment of the signals and chemical shifts presented in the NMR spectra. Table S1 shows the chemical shifts for carbons (^{13}C) and hydrogens (^{1}H) obtained experimentally from vitexin isolated in this work and their comparison with the literature. From the analysis of Table S1, it is possible to observe that the data obtained experimentally by NMR analysis of vitexin are very similar to those available in the literature [3,4], which is sufficient to confirm its structural determination.

Costa et al. [1] had already observed that *J. mutabilis* can be considered a promising source of vitexin, due to its relatively high yield for a high value-added natural product. This fact can be better understood when comparing the yield of vitexin from *J. mutabilis* with other plant sources, as is the case of the findings reported by Choo et al. [5], who reported the isolation of vitexin from the leaves of *Ficus deltoidea* Jack, Moraceae family,

with a yield of approximately 0.0001% in relation to dry plant. When analyzing the data mentioned here, it is clear that the vitexin yield of *J. mutabilis* is much higher than the yield of *Ficus deltoidei* (0.09%, about 900 times higher), demonstrating, once again, the importance of this caatinga species for obtaining vitexin.

One fact that can significantly alter the yield of vitexin obtained from a plant drug is both the extraction method and the solvents used, as can be seen in the work of Martino et al. [6]. The authors reported the content of vitexin extracted from leaves and flowers of *Crataegus monogyna* Jacq, Rosaceae family, by different methods (Soxhlet extraction, maceration, ultrasound-assisted extraction and microwave-assisted extraction). Among these methods, the one using microwave-assisted extraction with a solvent mixture composed of ethanol and water (1:1) was the one that obtained the highest vitexin content (1.198 ± 0.008 mg/g of vitexin in the extract), followed by the ultrasound-assisted extraction method with the same solvent mixture (1.100 ± 0.007 mg/g of vitexin in the extract). These findings reaffirm the importance of the choice of solvents and the extraction method in the extraction yield of natural products.

Table S1. Chemical shifts (δ) for vitexin.

**Position**	**^{13}C Vitexin (isolated)**	**^{1}H Vitexin (isolated)**	**^{13}C Vitexin (literature)** [3]	**^{1}H Vitexin (literature)** [3]
2	163.8	-	163.9	-
3	102.3	6.81	102.5	6.79
4	181.9	-	182.1	-
5	160.3	-	160.4	-
6	98.0	6.29	98.1	6.27
7	162.4	-	162.6	-
8	104.5	-	104.6	-
9	155.9	-	156.0	-
10	103.9	-	104.0	-
1′	121.5	-	121.6	-
2′	128.8	8.04	129.0	8.02
3′	115.7	6.89	115.8	6.87
4′	161.0	-	-	-
5′	115.7	6.89	115.8	6.87
6′	128.8	8.04	129.0	8.02
1′′	73.2	4.8	73.4	4.73
2′′	70.7	3.8	Ni	Ni
3′′	78.5	3.3	Ni	Ni
4′′	70.4	3.4	Ni	Ni
5′′	81.7	3.2	Ni	Ni
6′′	61.2	3.7 e 3.5	Ni	Ni
OH-5	-	13.2	-	13.17
OH-7	-	10.8	-	Ni
OH-4′	-	10.3	-	Ni

(Ni) Not identified.

References

1. Costa, E.C.; Menezes, P.M.N.; Silva, F.S.; Ribeiro, L.A. de A.; Rolim, L.A.; Araújo, E.C. da C.; Nunes, X.P. Jatropha Mutabilis , a New Source of Vitexin: HPLC Quantification and Pharmacological Evaluation. *Nat Prod Res* 2020, 1–4, doi:10.1080/14786419.2020.1837807.

2. Costa, E.C.; Menezes, P.M.N.; de Almeida, R.L.; Silva, F.S.; de Araújo Ribeiro, L.A.; de Silva, J.A.; de Oliveira, A.P.; da Cruz Araújo, E.C.; Rolim, L.A.; Nunes, X.P. Inclusion of Vitexin in β-Cyclodextrin:

Preparation, Characterization and Expectorant/Antitussive Activities. *Heliyon* 2020, *6*, e05461, doi:10.1016/j.heliyon.2020.e05461.

3. Ferreira, R.; Carvalho Jr., A.; Riger, C.; Castro, R.; Silva, T.; Carvalho, M. Constituintes Químicos e Atividade Antioxidante in Vivo de Flavonoides Isolados de Clusia Lanceolata (Clusiaceae). *Quim Nova* 2016, doi:10.21577/0100-4042.20160131.

4. Yu, X.-X.; Huang, J.-Y.; Xu, D.; Xie, Z.-Y.; Xie, Z.-S.; Xu, X.-J. Isolation and Purification of Orientin and Vitexin from *Trollius Chinensis* Bunge by High-Speed Counter-Current Chromatography. *Nat Prod Res* 2014, *28*, 674–676, doi:10.1080/14786419.2014.891111.

5. Choo, C.Y.; Sulong, N.Y.; Man, F.; Wong, T.W. Vitexin and Isovitexin from the Leaves of Ficus Deltoidea with In-Vivo α-Glucosidase Inhibition. *J Ethnopharmacol* 2012, *142*, 776–781, doi:10.1016/j.jep.2012.05.062.

6. Martino, E.; Collina, S.; Rossi, D.; Bazzoni, D.; Gaggeri, R.; Bracco, F.; Azzolina, O. Influence of the Extraction Mode on the Yield of Hyperoside, Vitexin and Vitexin-2″- *O* -rhamnoside from *Crataegus Monogyna* Jacq. (Hawthorn). *Phytochemical Analysis* 2008, *19*, 534–540, doi:10.1002/pca.1081.
